# CircHECTD1 Regulates Cell Proliferation and Migration by the miR-320-5p/SLC2A1 Axis in Glioblastoma Multiform

**DOI:** 10.3389/fonc.2021.666391

**Published:** 2021-05-17

**Authors:** Wen Li, Shanshan Wang, Boquan Shan, Xiang Cheng, Hui He, Jianbing Qin, Yi Tang, Heyan Zhao, Meiling Tian, Xinhua Zhang, Guohua Jin

**Affiliations:** Department of Anatomy and Neurobiology, Jiangsu Key Laboratory of Neuroregeneration, Collaborative Innovation Center of Neuroregeneration, Nantong University, Nantong, China

**Keywords:** circHECTD1, miR-320-5p, SLC2A1, proliferation, migration, GBM

## Abstract

Glioblastoma multiform (GBM) is the most common and malignant primary brain cancer in adults, and thus, novel potential therapeutic targets for diagnosis and treatment are urgently needed. Circular RNAs (circRNAs) are a class of widespread and diverse endogenous RNAs that have been suggested as potential critical mediators during progression of various tumors. In this study, we investigated the involvement of circHECTD1 in GBM progression. CircHECTD1 Lentivirus, miR-320-5p mimic, and SLC2A1 Lentivirus were transduced into cancer cells independently or together. circHECTD1, miR-320-5p, and SLC2A1 level were detected by qRT-PCR. Western blot and qRT-PCR were applied to measure the expression of SLC2A1, CyclinD1, CDK2, and PCNA. Flow cytometry, EdU, colony formation, Transwell and wound-healing assays were conducted to assess cell proliferation and migration. Luciferase reporter assays were performed to determine the effect of miR-320-5p on circHECTD1 or SLC2A1. Xenograft experiments were implemented to evaluate tumor growth *in vivo*. CircHECTD1 expression led to the promotion of proliferation and migration of GBM cells. In addition, circHECTD1 acted as a ceRNA to interact with miR-320-5p, which targeted the solute carrier family 2 member 1 (SLC2A1). In vivo experiments also revealed that circHECTD1 promoted tumor growth. Collectively, our findings showed that the circHECTD1-miR-320-5p-SLC2A1 regulatory pathway promoted the progression of GBM, suggesting that circHECTD1 may be a therapeutic target for GBM.

## Introduction

Glioblastoma multiform (GBM) is a malignant and aggressive primary brain tumor, characterized by rapid growth and invasion of the surrounding tissue (1). The existing treatments for glioblastoma are surgery, radiotherapy, and chemotherapy as an adjunct, which have limited success in increasing the overall survival of patients (2). In recent years, targeted agents have shown promise as novel therapies or as sensitizers to improve responses to traditional chemotherapy and radiation (3). Thus, identifying and understanding the key molecules associated with the malignant phenotype of GBM will yield new potential therapeutic targets.

Circular RNAs (circRNAs), one type of endogenous noncoding RNAs (ncRNAs), form a covalently closed continuous loop of single-stranded RNAs, which are ubiquitously present in mammalian cells (4). Previous studies have reported that circRNAs can act as competing endogenous RNAs (ceRNAs) by sharing common miRNA response elements to miRNA-targeted gene expression (5). It has been reported by many investigators that circRNAs interact with miRNA and transcription factors to regulate gene expression, which play essential roles in tumorigenesis and development (6). For example, circRNA-5692 inhibited the proliferation and migration of hepatocellular carcinoma by sponging oncogenic miR-328-5p, to enhance tumor suppressor DAB2IP expression (7). circPLEKHM3 was found to act as a ceRNA for miR-9, to upregulate BRCA1, DNAJB6 and KLF4, which subsequently activated the epithelial-mesenchymal transition, AKT1, and canonical Wnt/β-catenin signaling pathways (8). Song et al. (9) revealed a novel mechanism that hsa_circRNA_101996-miR-8075-TPX2 network contributed to cervical cancer proliferation, migration, and invasion.

In this study, we found that circHECTD1 effectively enhanced the proliferation and migration of GBM cells. Furthermore, we characterized the molecular mechanism of circHECTD1 and found that circHECTD1 acted as a ceRNA to sponge miR-320-5p to promote SLC2A1 expression and enhance the malignant behaviors of GBM. Overall, this study showed the effect of the circHECTD1-miR-320-5p-SLC2A1 axis in GBM, which highlighted potential novel targets for GBM treatment.

## Materials and Methods

### Animals and the Xenograft Tumor Model

Twenty-four four-week-old female athymic Balb/c nude mice were used. All animal experiments were carried out according to Institutional Animal Care Guidelines and were ethically approved by the Administration Committee of Experimental Animals, with the approval of Nantong University, Jiangsu, China. Approximately 1 × 10^7^ C6 cells were injected subcutaneously into each mouse. Six mice were used in each group. Mice were sacrificed and their tumor weights were measured after 3 weeks.

### Cell Culture

C6, U87 and HEK-293T cells lines were cultured in Dulbecco Modified Eagle Medium and Ham F-12 nutrient mixture (1:1, DMEM/F-12, Gibco, Carlsbad, CA, USA) supplemented with 10% fetal bovine serum (FBS, Gibco) and 1% penicillin/streptomycin (Sangon Biotech, Shanghai, China). All cells were incubated in a humidified atmosphere of 5% CO_2_ in air at 37°C.

### Transfection and Lentiviral Transduction

The transfection was performed when they were 60~80% confluent. The cells were transfected with the miR-320-5p/NC mimic or miR-320-5p/NC inhibitor (Ribobio, Guangzhou, China) using Lipofectamine 3000 (Invitrogen, Carlsbad, CA, USA) according to the manufacturer’s instructions. The circHECTD1 or SLC2A1 was subcloned into the lentivirus vector ((polyA-MCS-UBI)RV-SV40-EGFP-IRES-puromycin) to construct LV- circHECTD1 vector, while LV-NC was used as the negative control, and (hU6-MCS-Ubiquitin-EGFP-IRES-puromycin) to construct sh-circHECTD1 or sh-SLC2A1 vector, while sh-NC was used as the negative control. All lentiviruses were constructed by Genechem (Shanghai, China). After the lentiviruses were cultured for 48 h, the lentiviruses were removed and replaced with fresh medium. The cells were then screened by culturing in the presence of 2 μg/mL puromycin after transfection.

### RNA Isolation and RT-qPCR

The nuclear and cytoplasmic fractions were extracted using a Nucleoprotein Extraction kit (Invitrogen). Total RNA from cells and tissues were isolated using TRIzol Reagent (Vazyme Biotech, Nanjing, China) according to the manufacturer’s instructions. For mRNA expression analysis, 1 µg of RNA was used to synthesize cDNA using the HiScript Q RT SuperMix for qPCR (+gDNA wiper) (Vazyme Biotech, Nanjing, China), and the SYBR green method (Roche, Basel, Switzerland) was performed using the StepOnePlus™ real-time PCR system (Invitrogen). Primers for RT-qPCR analysis of circHECTD1, and linear mRNA are listed in **Table S1**.

For miRNA expression analysis, a miRcute Plus miRNA First-Strand cDNA Synthesis Kit (Tiangen Biotech, Beijing, China) and miRcute miRNA qPCR Detection Kit (Tiagen Biotech) were used. A total of 1 μg of total RNA was used according to the manufacturer’s protocol. Forward primers of miRNAs were obtained from Ribobio (Guangzhou, China), and the reverse primer was commercial, as supplied in a miRcute miRNA qPCR Detection Kit. The relative gene expression was calculated by comparing the cycle times for each target PCR. The target PCR Ct values were normalized by subtracting the internal control Ct value.

### Actinomycin D and RNase R Treatment

To block transcription, actinomycin D (1 μg/mL; APEXBIO Technology, Houston, TX, USA) or dimethyl sulfoxide (Sigma-Aldrich, St. Louis, MO, USA) as a negative control was added to the cell culture medium. For RNase R treatment, 1 μg of total RNA was incubated for 30 min at 37°C with or without 2 units/μg of RNase R and purified using the RNeasy MinElute cleaning Kit (Qiagen, Hilden, Germany).

### Fluorescence *In Situ* Hybridization (FISH)

FISH was performed in C6 cells, using a FISH kit purchased from RiboBio following the manufacturer’s protocol. After washing with PBS, the cells were fixed in 4% paraformaldehyde and then incubated in PBS with 0.3% Triton X-100. Before hybridization overnight at 4°C, the cells were incubated in the prehybridization solution for 30 min at 37°C. The next day, the cells were stained with Hoechst 33342 after washing with 4× saline sodium citrate (SSC) twice, 2× SSC once, 1× SSC once, and PBS once at 42°C. The images were then captured with a confocal fluorescent microscope (Olympus, Tokyo, Japan) and processed with identical settings for all images.

### Western Blotting

Total proteins were homogenized with RIPA lysis buffer containing 1 mM phenylmethylsulfonyl fluoride (Solarbio, Beijing, China). After determining the protein concentration, proteins in the supernatant were separated by 10% SDS-PAGE, transferred to polyvinylidene difluoride membranes and then blocked with 5% skim milk for 2 h. After incubating with primary antibodies overnight at 4°C, the membranes were incubated with goat anti-rabbit or anti-mouse horseradish peroxidase-linked secondary antibodies for 2 h. Bands on the membranes were visualized by enhanced chemiluminescence reagents (ECL; Bio-Rad, Hercules, CA, USA). Primary antibodies included anti-PCNA (1:1,000, Abcam, Cambridge, UK), anti-CyclinD1 (1:1,000, Abcam), anti-CDK2 (1:1,000, Abcam), anti-SLC2A1 (1:1,000, Abcam), and anti-β-actin (1:1,000, Abcam), anti-histone H3 (1:1,000, Abcam) and anti-β-tubulin (1:1,000, Abcam).

### Flow Cytometry for the Cell Cycle Assay

Cells in a culture dish were collected after 24 h, washed twice in ice-cold PBS, and fixed in 75% ice-cold ethanol in PBS. The cells were washed twice in PBS, then stained with BD Pharmingen™ PI/RNase Staining Buffer (BD Biosciences, San Diego, CA, USA) for 30 min at 4°C. The cell suspension was subjected to flow cytometry (BD Biosciences).

### Proliferation Assay

For the EdU assay, cells were seeded in plates and incubated with 50 mM of EdU using a Cell-Light EdU Apollo567 In Vitro Kit (Ribobio) for 2 h, and the cells were then stained for EdU as described in the manufacturer’s protocol. For the growth ability assay, 100 cells were trypsinized and plated into 100 mm dishes. After 10 days, the colonies were stained with 1.0% crystal violet for 30 min after fixation with 4% PFA for 15 min.

### Cell Migration Assay

The migration ability was measured in 24-well plates using Corning Transwell chambers with 8 μm filter membranes (Corning, Acton, MA, USA). The starved cells were plated in the upper chamber with serum-free medium and the serum medium was placed in the lower chamber. The remaining cells on the upper surface were wiped with a cotton swab after 24 h. Afterwards, the cells on the lower surface of the filter were fixed with 4% paraformaldehyde and stained with 1% crystal violet for 30 min. For the wound-healing assay, the cells were cultured in 6-well plates until they were a single confluent layer. The cell monolayer was scraped in three straight lines with a 1,000 μl pipette tip to create “scratches”. Cell debris was removed by PBS washing, and fresh medium was added.

### Luciferase Reporter Assay

The luciferase reporter vectors were constructed by Genechem. The circHECTD1 and SLC2A13’-UTR, which included miR-320-5p seed binding sites, was cloned into the pGL3-promotor vector to construct the wide type (WT) vector, and the predicted seed zones with miR-320-5p were replaced by nonsense sequences in mutation type (MuT) vector. In the luciferase reporter assay, HEK-293 cells plated in a 24-well plate were co-transfected with 100 ng plasmid and 100 ng luciferase construct. Luciferase and Renilla signals were measured 72 h after transfection using the Dual Luciferase Reporter Assay Kit (Promega, Madison, WI, USA) according to a protocol provided by the manufacturer.

### Immunohistochemistry

Coronal sections were blocked in PBS containing 0.3% Triton X-100 and 10% goat serum for 2 h, and then incubated with rabbit anti-SLC2A1 (1:1,000, Abcam) overnight at 4°C. After rinsing in PBS, the sections underwent color development with 3, 3′-diaminobenzidine (BD Pharmingen, NY, NY, USA) and hematoxylin (Vector Laboratories, Burlingame, CA, USA) counterstaining.

### Statistical Analysis

The quantitative results were presented as the mean ± SEM based on at least three independent experiments. All data were analyzed by Prism software, version 6.00 (GraphPad Software, La Jolla, CA, USA). Experiments with two experimental groups were evaluated using an unpaired Student’s two-tailed *t*-test. In experiments with more than two experimental groups, one-way analysis of variance was used. The results were considered statistically significant when ^*^ *P* < 0.05; ^**^ *P* < 0.01; or ^***^ *P* < 0.001.

## Results

### Characteristics of the circHECTD1 Circular RNA

**Figure 1:**
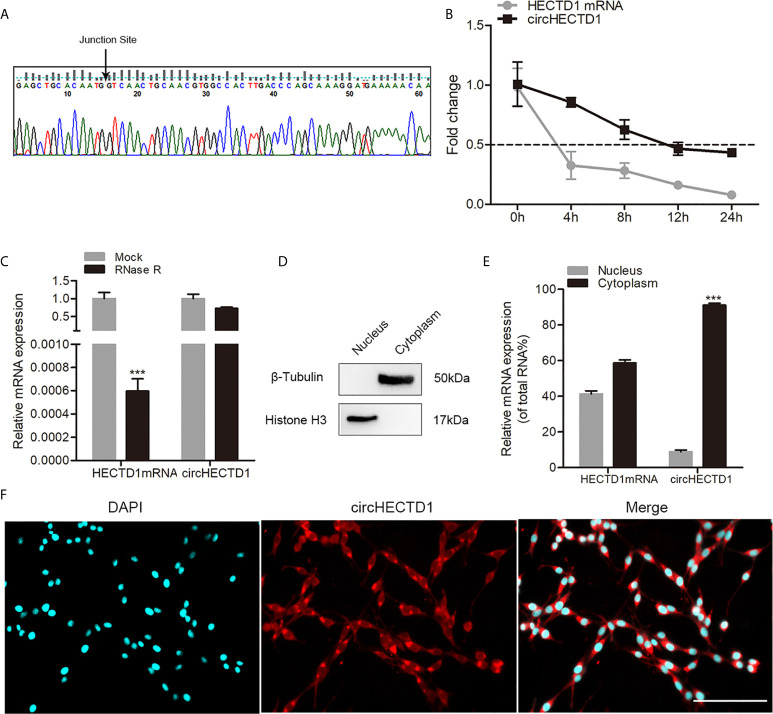
Characteristics of circHECTD1**. (A)** The presence of circHECTD1 was validated by RT-qPCR followed by Sanger sequencing. **(B)** RT-qPCR analysis of the abundance of circHECTD1 and HECTD1 mRNA treated with actinomycin D at the indicated time points. **(C)** RT-qPCR analysis of the abundance of circHECTD1 and HECTD1 mRNA treated with RNase R (RNase R) or without RNase R (Mock). **(D)** Western blot analysis of cytoplasmic (β-tubulin) and nuclear (histone H3) marker protein expression. **(E)** RT-qPCR data indicating the abundance of circHECTD1 and HECTD1 mRNA in either the cytoplasm or nucleus. **(F)** Fluorescence *in situ* hybridization assay showing the location of circHECTD1. The nuclei were stained with Hoechst dye. Bar = 200μm. Each bar represents the mean of three independent experiments. ^***^ *P* < 0.001.

To validate the existence of circHECTD1 in C6 cells, junction primers were designed to confirm the size and sequence of an amplified PCR product, followed by Sanger sequencing (**Figure 1A**). Furthermore, to investigate the stability of circHECTD1 in cells, we extracted the total RNA treated with actinomycin D, an RNA synthesis inhibitor. The results showed that the half-life of circHECTD1 was approximately 12 h, whereas the linear HECTD1 mRNA exhibited a half-life that was less than 4 h (**Figure 1B**). The expression of circHECTD1 was resistant to digestion with RNase R exonuclease, suggesting that the studied RNA species was likely circular in form (**Figure 1C**). In addition, nucleoplasmic separation and the FISH assay showed that circHECTD1 was predominantly localized in the cytoplasm (**Figures 1E, F**). Fraction purity was confirmed by western blot using antibodies against nuclear (histone H3) and cytoplasmic (β-tubulin) marker proteins (**Figure 1D**).

### CircHECTD1 Regulated the Proliferation and Migration of GBM Cells

**Figure 2:**
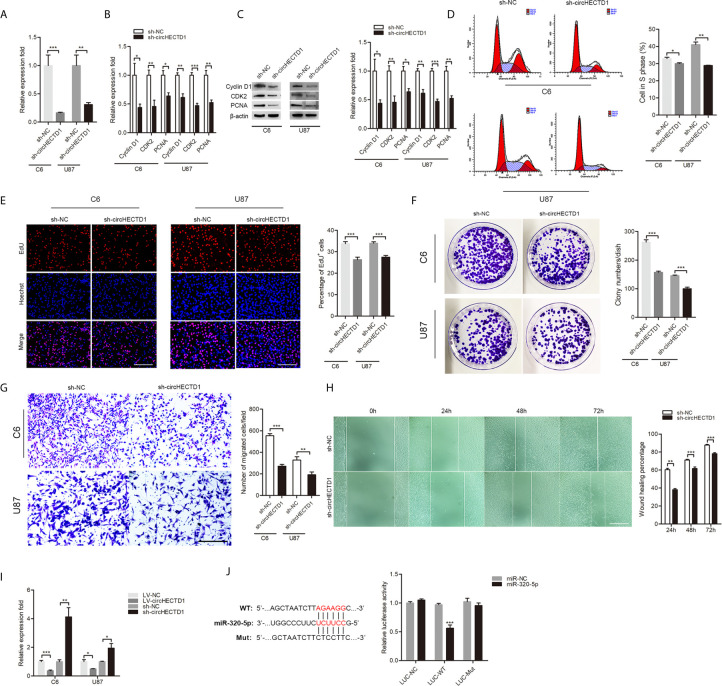
CircHECTD1 regulated the cell proliferation and migration. **(A)** RT-qPCR analysis of circHECTD1 expression. **(B, C)** The expression of proliferation marker genes, CyclinD1, CDK2, and PCNA, were measured by RT-qPCR and western blot. **(D)** Flow cytometric analysis of the indicated cells transfected with the normal control or circHECTD1. **(E)** Representative micrographs and quantification of EdU incorporating-cells. Nuclei were stained with Hoechst dye. Bar = 200μm. **(F)** Representative micrographs and quantification of crystal violet-stained cell colonies. **(G)** The cells migrating to the lower surface of the membrane were photographed and counted. Bar = 200μm. **(H)** Photographs were taken when the wound was created at 0 h, 24 h, 48 h, and 72 h in C6 cells, and the percentages of cells migrating into the wound were counted. Bar = 400μm. **(I)** RT-qPCR analysis depicts the expression of miR-320-5p in the control and circHECTD1 group. **(J)** Luciferase activity of LUC-circHECTD1 wild-type and mutant in C6 cells transfected with the miR-320-5p mimic and negative control mimic. The data represent the mean ± SEM of three independent experiments. ^*^P < 0.05*;* ^**^ *P* < 0.01; ^***^ *P* < 0.001.

To determine the functional association of circHECTD1 in GBM cell lines, lentivirus was transduced into cells, and expression of circHECTD1 was confirmed by RT-qPCR (**Figure 2A**). We found that circHECTD1 knockdown significantly decreased the expression of proliferation-related markers, including CyclinD1, CDK2, and PCNA at both the mRNA and protein levels (**Figures 2B, C**). Flow cytometry assays showed that circHECTD1 knockdown significantly decreased the percentage of cells in S phase (**Figure 2D**). Similarly, using an EdU incorporation assay, we found that circHECTD1 knockdown repressed the mitotic activity (**Figure 2E**). Consistent with the abovementioned results, we found that circHECTD1 suppression decreased the colony numbers (**Figure 2F**). We further examined the effect of circHECTD1 on cell migration using the Transwell and wound-healing assays. The results showed that the migration of cells was significantly decreased after circHECTD1 knockdown (**Figures 2G, H**). Taken together, these results verified the oncogenic roles of circHECTD1 in GBM.

### CircHECTD1 Directly Bound to miR-320-5p and Suppressed miR-320-5p Activity

We used the online bioinformatics database (RNAhybrid) (10) to predict the interacting miRNAs of circHECTD1. Among the candidate miRNAs, miR-320-5p was of particular interest. miR-320 has been described as a tumor suppressor in tumor pathogenesis and progression (11–13). Overexpression of circHECTD1 significantly resulted in a significant decrease of miR-320-5p, whereas circHECTD1 knockdown significantly increased the relative abundance of miR-320-5p (**Figure 2I**). The luciferase assay showed that overexpression of miR-320-5p remarkably repressed luciferase activity in wild-type circHECTD1, but not in the mutant form (**Figure 2J**). Collectively, these results demonstrated that miR-320-5p was an actual target of circHECTD1.

### The circHECTD1-miR-320-5p Axis Was Crucial for Cell Proliferation and Migration in GBM

**Figure 3:**
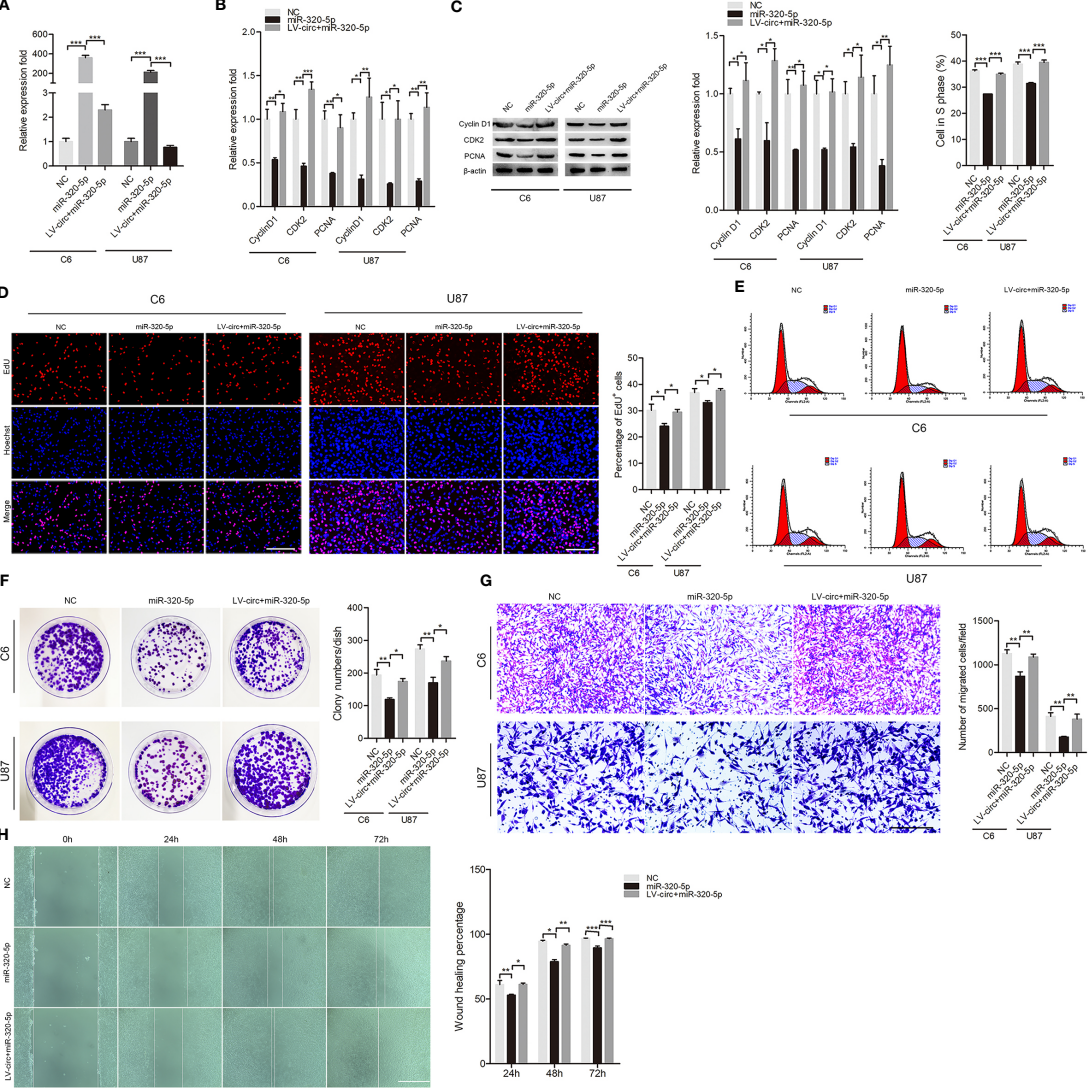
The effect of miR-320-5p on cell proliferation and migration. **(A)** RT-qPCR analysis of miR-320-5p expression. **(B, C)** The mRNA and protein levels of proliferation marker genes were measured by RT-qPCR and western blotting. **(D)** After 24 h, cell proliferation was detected by the EdU assay. The nuclei were stained with Hoechst dye. Bar = 200μm. **(E)** Flow cytometric analysis of the cell cycle distribution. **(F)** Representative micrographs and quantification of crystal violet-stained cell colonies. **(G)** Transwell assay results of the migration abilities were photographed and counted. Bar = 200μm. **(H)** After transfection, photographs were taken when the wound was created at 0 h, 24 h, 48h, and 72 h in C6 cells, and the percentages of migrated cells were calculated. Bar = 400μm. The data represent the mean ± SEM of three independent experiments. ^*^ *P* < 0.05; ^**^ *P* < 0.01; ^***^ *P* < 0.001 compared with the control.

To investigate whether the circHECTD1-miR-320-5p interaction regulated the function of GBM cell lines, we treated cells with miR-320-5p or circHECTD1, and the expression was verified by RT-PCR (**Figure 3A**). To investigate the functional role of circHECTD1-miR-320-5p in the proliferation of cells, we performed RT-PCR analysis, western blotting, the EdU assay, flow cytometry, and the colony formation assay. We detected the expression of CyclinD1, CDK2, and PCNA and found that miR-320-5p decreased the expression levels of these genes, but when co-transfected with circHECTD1, the expression of these genes increased (**Figures 3B, C**). Compared with the control, transfection using miR-320-5p significantly decreased the number of cells, whereas circHECTD1 significantly rescued cell phenotypes by increasing cell proliferation (**Figure 3D**). The results of flow cytometry were similar (**Figures 3E**). Consistently, colony formation assays revealed that miR-320-5p decreased the growth rate of cells, and circHECTD1 had the opposite effect on cell proliferation (**Figure 3F**). To investigate whether the circHECTD1-miR-320-5p axis regulated the migration of cancer cells, we transfected cells with miR-320-5p mimic after overexpression of circHECTD1. Compared with the control, the results showed that transfection with miR-320-5p significantly decreased cell migration, whereas circHECTD1 significantly promoted the migration of cells (**Figures 3G, H**). Together, these results suggested that circHECTD1 may play an oncogenic role in GBM cells by suppressing miR-320-5p activity.

### The miR-320-5p Directly Targeted SLC2A1

**Figure 4:**
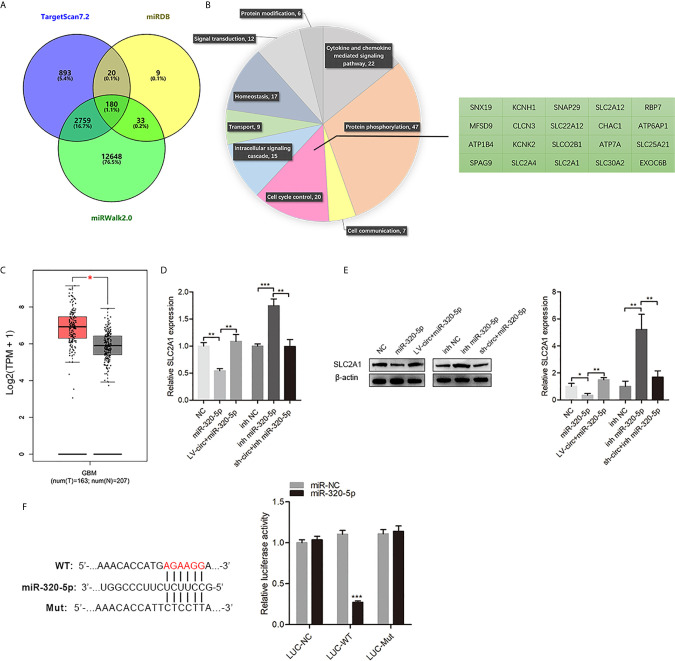
SLC2A1 was a direct target of miR-320-5p. **(A)** The predicted target genes of miR-320-5p were collected from three target prediction algorithms (miRWalk, TargetScan, and miRDB). **(B)** The predicted target genes of miR-320-5p were analyzed according to the PANTHER classification system. **(C)** The expression of SLC2A1 in glioblastoma multiform clinical samples. **(D, E)** RT-qPCR assays and western blot analyses were used to determine changes in SLC2A1 expression levels for the control, miR-320-5p, and (or) circHECTD1-treated sets. **(F)** The luciferase reporter assay of the indicated cells co-transfected with the luciferase-SLC2A1 3′-UTR fusion and miR-320-5p or vector, 72 h post-transfection. Data are presented as the mean ± SEM of three independent experiments. ^*^ *P* < 0.05; ^**^ *P* < 0.01; ^***^ *P* < 0.001.

To identify miR-320-5p-mediated downstream regulators in GBM cells, we identified and screened potential targets of miR-320-5p by three target prediction algorithms (miRWalk, TargetScan, and miRDB) (14, 15) (**Figure 4A**). The 180 picks from the algorithms were further analyzed according to the PANTHER classification system, in which 20 genes were clustered in the cell cycle control (**Figure 4B**). According to GEPIA (https://gepia.cancer-pku.cn/) (16), we found that SLC2A1 was highly expressed in GBM compared with normal samples (**Figure 4C**). The miR-320-5p in GBM cells resulted in a significant decrease of SLC2A1, but circHECTD1 somewhat relieved this effect (**Figures 4D, E**). As shown in **Figure 4F**, overexpressing miR-320-5p efficiently reduced the expression of the luciferase reporter in the wild-type group but did not have the same effect in the mutant (mut) group. Collectively, these results showed that SLC2A1 was the direct target of miR-320-5p, which could suppress SLC2A1 function in GBM cells.

### CircHECTD1 Promoted the Proliferation and Migration by Sponging miR-320-5p to Regulate SLC2A1

**Figure 5:**
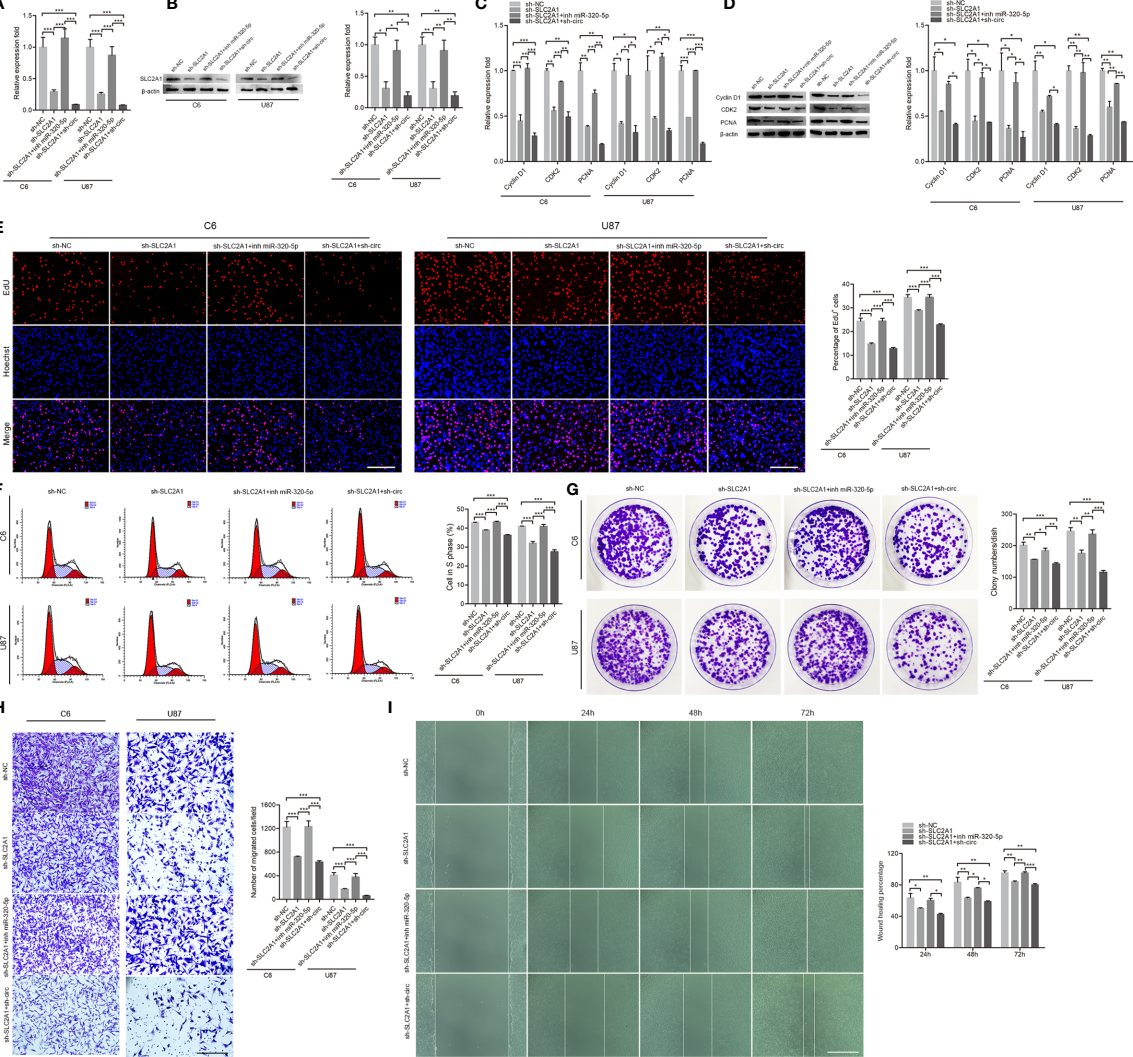
CircHECTD1 controlled cell proliferation and migration by binding miR-320-5p to regulate SLC2A1 expression. **(A, B)** RT-qPCR analysis and western blot of the relative abundance of SLC2A1 expression. **(C, D)** RT-qPCR and western blot analyses of the proliferation marker genes. **(E)** Representative micrographs and quantification of the EdU assay. Nuclei were stained with Hoechst dye. Bar = 200μm. **(F)** Flow cytometric measurement of the cell cycle distribution. **(G)** Representative micrographs and quantification of crystal violet-stained cell colonies. **(H)** The cellular migration was determined by the Transwell assay. Bar = 200μm. **(I)** After transfection, photographs were taken when the wound was created at 0 h, 24 h, 48 h, and 72 h in C6 cells, and the percentages of migrated cells were calculated. Bar = 400 μm. The data represent the mean ± SEM of three independent experiments. ^*^ *P* < 0.05; ^**^ *P* < 0.01; ^***^ *P* < 0.001 compared with the control.

To characterize the roles of SLC2A1 in the proliferation and migration of GBM cells, we knockdown SLC2A1 (**Figures 5A, B**). We performed proliferation and migration assays using cells transfected with SLC2A1, together with miR-320-5p or circHECTD1. As shown in **Figures 5C–I**, the results demonstrated that SLC2A1 knockdown remarkably suppressed cell proliferation and migration, whereas downregulation of miR-320-5p significantly increased cell proliferation and migration. In addition, transfection with SLC2A1 after knockdown of circHECTD1 showed further alleviated cell proliferation and migration. Overall, these results demonstrated that the circHECTD1-miR-320-5p-SLC2A1 axis was an important regulator in the progression of GBM.

### The Therapeutic Potential of circHECTD1 *In Vivo*

**Figure 6:**
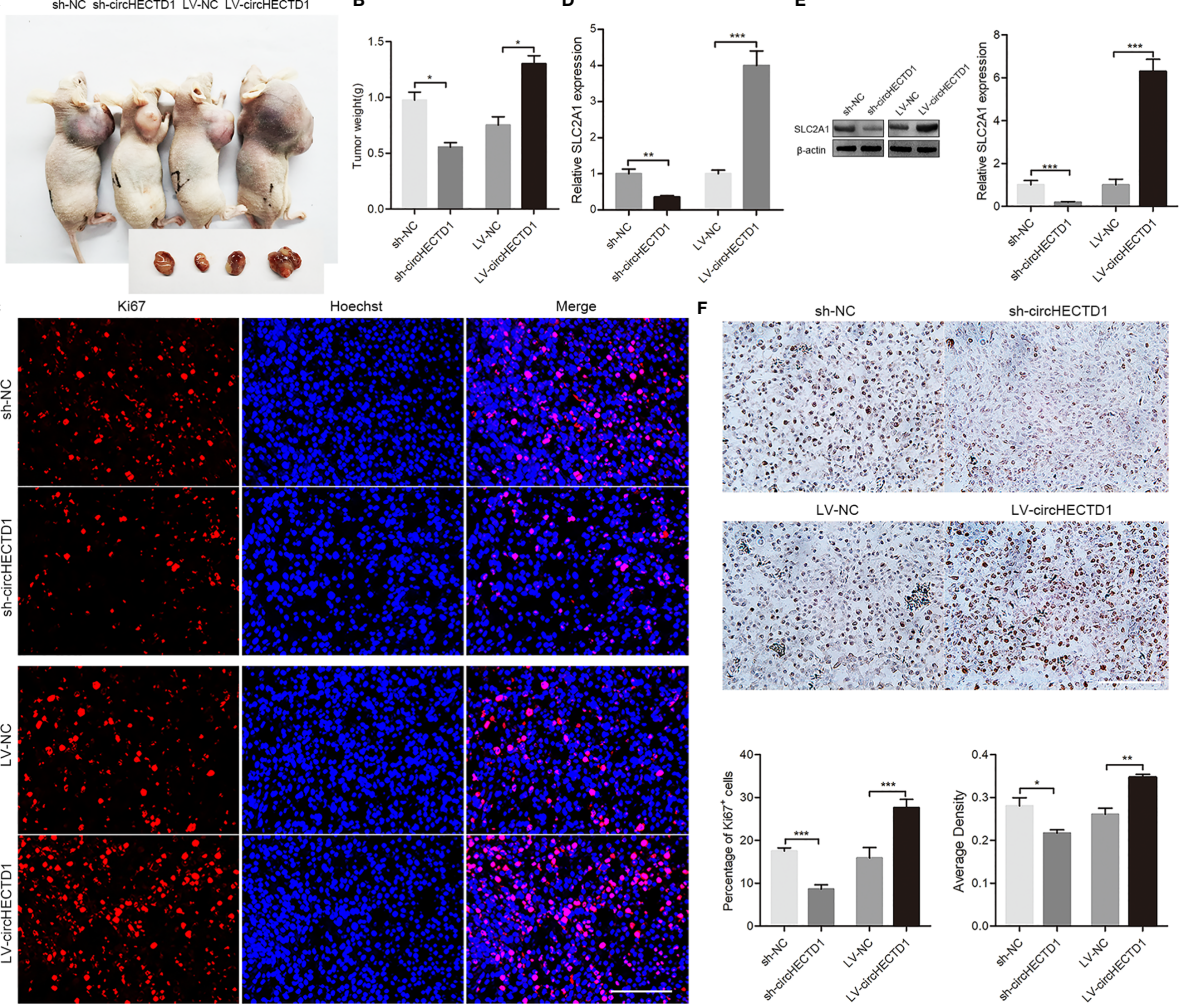
CircHECTD1 enhanced gliobastoma multiform growth *in vivo.* **(A)** Representative images in immunodeficient mice. **(B)** The tumor weights of immunodeficient mice injected with C6 cells. **(C)** Representative micrographs and quantification of Ki67 positive cells after transfection with circHECTD1 or the normal control. The nuclei were stained with Hoechst dye. Bar = 200μm. **(D, E)** The expression of SLC2A1 were detected by RT-qPCR and Western blot analyses. **(F)** Representative images and quantification of immunohistochemical staining for SLC2A1. The values are the mean ± SEM for three biological replicates. ^*^ *P* < 0.05; ^**^ *P* < 0.01; ^***^ *P* < 0.001.

To further confirm the role of circHECTD1 *in vivo*, we established a nude mice xenograft model by subcutaneous inoculation of C6 cells stably transfected with circHECTD1 or sh-circHECTD1. We observed that tumor volumes and weights were significantly smaller in the sh-circHECTD1 group than those in the control group, whereas they were significantly larger in the group stably expressing circHECTD1 (**Figures 6A, B**). Immunofluorescence staining showed that circHECTD1 knockdown significantly decreased the number of Ki67 positive cells, whereas it was significantly increased after circHECTD1 overexpression (**Figure 6C**). Analysis by RT-qPCR and western blotting showed that the expression levels of SLC2A1 were significantly decreased in tumor tissues after circHECTD1 knockdown. Conversely, upregulation of circHECTD1 significantly increased the expression levels of SLC2A1 (**Figures 6D, E**). The results of the immunohistochemical analyses were similar (**Figure 6F**). Together, these results further validated our findings that circHECTD1 aggravated the tumorigenicity *in vivo*.

## Discussion

Circular RNAs (circRNAs) are a recently discovered large group of RNAs, which are deregulated in pathological phenotypes from cancer to degenerative conditions (17, 18). For example, Song et al. (19) reported that circHMCU promoted proliferation and metastasis of breast cancer by sponging the let-7 family, and can be used as a novel biomarker in diagnosis and prognosis. Chen et al. (20) showed that circRNA_100290 enhanced cell cycle progression by increasing CDK6 expression through sponging miR-29b family members. Sang et al. (21) revealed that hsa_circ_0025202 could suppress tumor growth and enhance tamoxifen efficacy *via* the miR-182-5p/FOXO3a axis. These reports illustrate the key role of circRNAs as regulators of critical signaling pathways, as well as their clinical value as novel biomarkers.

CircHECTD1 is derived from the *HECTD1* gene, and HECTD1 is widely expressed in a range of human and murine primary cells and cell lines, including macrophages, neuronal cells, and insulin secreting β-cells (22). Intervention of circHECTD1 significantly inhibits astrocyte activation with amelioration of cerebral infarction, indicating that circHECTD1 can be regarded as a novel biomarker for stroke (23). Cai et al. (24) reported that circHECTD1 was the most significantly upregulated circRNAs in gastric cancer, and upregulated circHECTD1 facilitates glutaminolysis by modulating the miR-1256/USP5 axis, thereby promoting gastric cancer progression. Jiang et al. (25) revealed that circHECTD1 facilitated hepatocellular carcinoma progression by sponging miR-485-5p to up-regulate of the mucin 1 level. In the present study, we showed that circHECTD1 facilitated the proliferation and migration *in vitro* and *in vivo*, suggesting that circHECTD1 may serve as an oncogene in GBM.

Recent studies have revealed that circRNAs play important roles in regulating gene expression at transcriptional or posttranscriptional levels, by acting as a miRNA sponge, transcription regulator, and protein decoy (26). Given that circHECTD1 is located predominantly in the cytoplasm, we speculated that circHECTD1 might directly target certain miRNAs. Our results indicated that miR-320-5p directly bound to circHECTD1, and the luciferase assay further showed that circHECTD1 and miR-320-5p physically bound to each other. Numerous studies have identified miR-320 was a tumor suppressor in tumor pathogenesis and progression. For example, Li et al. (27) found that miR-320 supressed cell proliferation, cycle and invasion through targeting TWIST1 in ovarian cancer. Zhang et al. (28) revealed that miR-320 was a tumor suppressor in breast cancer, and that the miR-320/ELF3 axis regulated tumor progression *via* the PI3K/AKT signaling pathway. Pan et al. (29) reported miR-320 suppressed C6 cell proliferation and induced cell cycle arrest and apoptosis through the PBX3/Raf-1/MAPK axis. In our study, overexpression of miR-320-5p suppressed cell proliferation and migration, whereas circHECTD1 significantly rescued cell phenotypes. Collectively, these results suggested that circHECTD1 bound directly to miR-320-5p and suppressed miR-320-5p activity.

To further elucidate the mechanisms underlying circHECTD1/miR-320-5p-mediated GBM progression, we explored the downstream effectors. According to the PANTHER classification system, we found that 20 genes are clustered in cell cycle control, of which SLC2A1 was highly expressed in GBM compared with normal samples. High SLC2A1 expression correlates with poor prognosis in patients with surgically resected lung adenocarcinoma (LUAD), which suggests that SLC2A1 is a promising biomarker for LUAD (30). Song et al. (31) found that HNF4A increased the expression of hexokinase 2 and SLC2A1, resulting in transactivation of CTCF and transcriptional alteration of HNF4A and other genes associated with tumor progression. Ding et al. (32) discovered that miR-148b inhibited glycolysis in gastric cancer through targeting SLC2A1. Based on these reports, we focused on SLC2A1, and indicated that circHECTD1 positively regulated the expression of SLC2A1 *via* sponging miR-320-5p during the malignant behavior of GBM cells.

## Conclusions

Our results revealed that circHECTD1 acted as an oncogene that promoted cell proliferation and migration. Mechanistically, circHECTD1 bound to miR-320-5p to regulate the endogenous expression of SLC2A1. Our study therefore provides a solid basis to develop a better understanding of GBM, as well as to identify potential targets for anti-cancer therapy in GBM.

## Data Availability Statement 

The original contributions presented in the study are included in the article/**Supplementary Material**, further inquiries can be directed to the corresponding authors.

## Ethics Statement

The animal study was reviewed and approved by Nantong University. All authors agree to publish this manuscript.

## Author Contributions

WL, SW and BS conceived the experiments. WL performed the experiments with help from XC, HH, JQ, YT, HZ, and MT. WL and GJ analyzed the data. WL, XZ, and GJ wrote the manuscript. All authors contributed to the article and approved the submitted version.

## Funding

Contract grant sponsor: Graduate Scientific Research Innovation Program of Jiangsu Province; Contract grant number: KYCX19 2066. Contract grant sponsor: a Project Funded by the Priority Academic Program Development (PAPD) of Jiangsu Higher Education institutions. Contract grant number: 03081023.

## Conflict of Interest

The authors declare that the research was conducted in the absence of any commercial or financial relationships that could be construed as a potential conflict of interest.
